# Supplementary material for: Clinical Implications of Multi-Drug Resistant Organisms’ Gastrointestinal Colonization in an Internal Medicine Ward: The Pandora’s Box
Source: J Clin Med. 2022 May 14;11(10):2770. doi: 10.3390/jcm11102770 (PMC9144986; doi:10.3390/jcm11102770)
Supplement: Supplementary file 1 [file jcm-11-02770-s001.zip › jcm-1646927-supplementary.pdf]

Supplementary Table S1. Reason for hospitalization in general population and comparison between patients with or without RS+ for MDRO.

Reason for hospitalization (n, %)	General population (454)	RS positive (77)	RS negative (377)	p
**Infectious diseases**	123 (27.1%)	28 (36.4%)	95 (25.2%)	<0.05
**Atherothrombotic diseases**	46 (10.1%)	3 (3.9%)	43 (11.4%)	<0.05
**Chronic diseases**	164 (36.1%)	38 (49.4%)	126 (33.4)	<0.01
**Other**	120 (24.4%)	8 (10.4%)	112 (29.7%)	<0.001

RS: Rectal Swab.

Supplementary Table S2. Univariate and multivariate analysis for in-hospital mortality.

Variable	In-hospital mortality (30)	Controls (424)	Univariate analysis	Multivariate analysis	
			p	OR	CI
**Male gender (n, %)**	17 (56.6)	232 (55.8)	0.85		
**Age (years; mean±SD)**	82.8±8.45	72.7±16.5	**<0.01**		
**Age > 75 aa (n, %)**	24 (80.0)	220 (51.9)	**0.003**	**3.28**	**1.26-8.50**
**Admission for ID (n, %)**	11 (36.7)	112 (26.4)	0.22		
**Admission for ATD (n, %)**	3 (10.0)	28 (6.6)	0.48		
**Admission for CDr (n, %)**	9 (30.0)	155 (36.6)	0.47		
**Admission for other reason (n, %)**	7 (23.3)	113 (26.6)	0.69		
**Solid active neoplasm (n, %)**	6 (20.7)	100 (23.6)	0.72		
**≥ 2 comorbidities (n, %)**	20 (66.7)	213 (50.2)	0.08		
**Immunomodulatory therapy (n, %)**	11 (36.7)	113 (26.6)	0.23		
**RS+ for MDRO (n, %)**	5 (16.7)	72 (17.0)	0.95		
**Sepsis during hospitalization (n, %)**	5 (16.7)	28 (6.6)	**0.04**		

ID: Infectious diseases; ATD: Atherothrombotic diseases; CDr: Exacerbation of chronic diseases; RS: Rectal Swab; MDRO: Multidrug resistant organism.

Supplementary Table S3. Univariate and multivariate analysis for 30-day mortality.

Variable	30 days mortality (49)	Controls (404)	Univariate analysis	Multivariate analysis	
			p	OR	CI
**Male gender (n, %)**	27 (55.1)	222 (54.9)	0.98		
**Age (years; mean±SD)**	81.6±8.6	72.4±16.7	**<0.001**		
**Age > 75 aa (n, %)**	35 (71.4)	209 (51.6)	**0.01**	**2.02**	**1.01-4.00**
**Admission for ID (n, %)**	16 (32.6)	107 (26.4)	0.35		
**Admission for ATD (n, %)**	5 (10.2)	26 (6.42)	0.32		
**Admission for CDr (n, %)**	19 (38.8)	145 (35.8)	0.68		
**Admission for other reason (n, %)**	9 (18.4)	111 (27.4)	0.17		
**Solid active neoplasm (n, %)**	11 (22.0)	95 (23.5)	0.92		
**≥ 2 comorbidities (n, %)**	32 (65.3)	201 (49.6)	**0.04**		
**Immunomodulatory therapy (n, %)**	17 (34.7)	107 (26.4)	0.22		
**RS+ for MDRO (n, %)**	11 (22.4)	66 (16.3)	0.28		
**Sepsis during hospitalization (n, %)**	7 (14.3)	26 (6.42)	**0.04**		

ID: Infectious diseases; ATD: Atherothrombotic diseases; CDr: Exacerbation of chronic diseases; RS: Rectal Swab; MDRO: Multidrug resistant organism.
